# In Vitro Structural Characteristics and Antioxidant and Expectorant Activities of Polysaccharides from *Citri grandis fructus immaturus*

**DOI:** 10.3390/antiox14040491

**Published:** 2025-04-18

**Authors:** Jingwen Li, Suifen Mo, Yingshan Feng, Yan Xiang, Chen Ni, Qing Luo, Jing Zhou, Yujia Wang, Ruoting Zhan, Ping Yan

**Affiliations:** 1 School of Chinese Materia Medica, Guangzhou University of Chinese Medicine, Guangzhou 510006, China; 20221110107@stu.gzucm.edu.cn (J.L.); 20201110687@stu.gzucm.edu.cn (S.M.); 20221120278@stu.gzucm.edu.cn (Y.F.); 20231120285@stu.gzucm.edu.cn (Y.X.); 020710@gzucm.edu.cn (C.N.); 20221120287@stu.gzucm.edu.cn (Q.L.); 20231110115@stu.gzucm.edu.cn (J.Z.); 20231120273@stu.gzucm.edu.cn (Y.W.); 2 Key Laboratory of Chinese Medicinal Resource from Lingnan, Guangzhou University of Chinese Medicine, Ministry of Education, Guangzhou 510006, China; 3 Joint Laboratory of National Engineering Research Center for the Pharmaceutics of Traditional Chinese Medicines, Guangzhou 510006, China

**Keywords:** antioxidant, expectorant, polysaccharide, *Citri grandis fructus immaturus*, traditional medicine

## Abstract

The aim of this study was to investigate the structural characteristics of four polysaccharides derived from *Citri grandis fructus immaturus* and their antioxidant and expectorant activities. ECP1 fraction passing through a 500 kDa dialysis bag (ECP1A) and ECP2 fraction retained in a 300 kDa dialysis bag (ECP2B) had molecular weights of 340 and 1217 kDa, respectively. All four polysaccharides were composed of six monosaccharides, including l-rhamnose, d-arabinose, d-xylose, d-mannose, d-glucose, and d-galactose, with molar ratios of 1.99:52.38:6.99:2.64:5.15:31.15 for ECP1A and 1.54:65.13:6.34:2.51:3.58:22.07 for ECP2B. ECP1A had an α/β-glucopyranose ring, and the glycosyl groups were linked mainly by 1→4, 1→2, or 1→6 glycosidic bonds. It likely adopted a single-stranded helical conformation. ECP2B had a β-glucopyranose ring, and the glycosyl groups were linked mainly by 1→4, 1→2, or 1→6 glycosidic bonds. Furthermore, in vitro experiments showed that ECP1A displayed excellent antioxidant activity (IC_50_ = 0.4614 mg/mL). ECP2B significantly inhibited MUC5AC mucin content expression in the mucus hypersecretion model of BEAS-2B cells, thus exerting an expectorant effect. A significant negative correlation was observed between the molecular weight of *Citri grandis fructus immaturus* polysaccharides and their antioxidant activity, and the uronic acid and d-arabinose contents of these polysaccharides exhibited strong negative trends with both antioxidant and expectorant activities. This study shows the potential for developing and utilizing polysaccharides from *Citri grandis fructus immaturus* as an antioxidant and expectorant agent.

## 1. Introduction

Plant polysaccharides are naturally occurring macromolecules that have gained significant attention due to their diverse pharmacological and physiological properties, including antioxidant, hypoglycemic, antitumor [1,2], immunomodulatory [3], anti-inflammatory, hypolipidemic [4,5], and anti-fatigue properties [6], as well as low toxicity and mild side effects. Furthermore, it has been reported that the spatial molecular structure of polysaccharides is intimately associated with their biological activities [7].

*Citri Grandis Exocarpium* is the unripe or nearly ripe dried exocarp of *Citrus grandis* ‘Tomentosa’ or *Citrus grandis* (L.) Osbeck of the Rutaceae family [8], which is a national geographical indication product of China, mainly produced in Huazhou City, Guangdong Province, and it has been cultivated for more than 1600 years. *Citri grandis fructus immaturus* is the dried young fruit of *Citrus grandis* ‘Tomentosa’ in the Rutaceae family [9]. As a traditional Chinese herbal medicine, *Citri Grandis Exocarpium* is clinically used to treat bronchitis, asthma, wind-cold cough, phlegm, chronic cough, and other common illnesses [10], and it has both medicinal and nutritional applications. It contains flavonoids, polysaccharides, essential oils, and other active ingredients [11,12]. It has been found that *Citri Grandis Exocarpium* polysaccharides have significant pharmacological effects, including antitussive, expectorant [13], antioxidant, anti-fatigue [14], antidementia [15], hypoglycemic, hypolipidemic [16], and anti-inflammatory effects. In addition to its medical use, *Citri Grandis Exocarpium* can be utilized to make meals and dietary supplements. Pharmacological experiments have shown that *Citri Grandis Exocarpium* polysaccharides have a significant effect on relieving cough and dissolving phlegm, and the First Affiliated Hospital of Sun Yat-sen University processed its polysaccharides into granules, which clinical experiments have revealed to have a therapeutic effect on chronic obstructive pulmonary emphysema and chronic bronchitis [17]. Few studies have investigated *Citri Grandis Exocarpium* polysaccharides, with most existing research focusing on the pharmacological activities of crude polysaccharides while lacking comprehensive structural characterization [13,14,15]. Research on the structural characterization of *Citri Grandis Exocarpium* polysaccharides remains limited. Cheng et al. conducted preliminary structural analyses limited to monosaccharide composition and Fourier transform infrared (FT-IR) spectroscopy [18]. Furthermore, the correlation between the *Citri Grandis Exocarpium* polysaccharide structure and their antioxidant and expectorant activities remains unclear, requiring further systematic investigation.

In this study, the crude polysaccharides of *Citri grandis fructus immaturus* were obtained via water extraction and ethanol deposits, deproteinized using the Sevage method, and purified using a diethylaminoethyl (DEAE) Sepharose fast flow chromatography column and dialysis bag with different molecular weights (Mw) to obtain the four polysaccharides. Gas chromatography-mass spectrometry (GC-MS) and periodate oxidation-Smith degradation were used to systematically investigate the chemical compositions and structural properties of the four polysaccharide fractions, and their antioxidant activities were evaluated. Furthermore, the expectorant activity of the four polysaccharides was estimated by using a mucus hypersecretion model with BEAS-2B cells.

## 2. Materials and Methods

### 2.1. Materials and Reagents

*Citri grandis fructus immaturus* was purchased in Huazhou City, Guangdong Province, China. Phenol, concentrated sulfuric acid, petroleum ether, anhydrous ethanol, trichloroacetic acid, and ethyl acetate were purchased from Tianjin Zhiyuan Chemical Reagent Co., Ltd. (Tianjin, China). DEAE Sepharose fast flow anion exchange resin was provided by GE health care company (Aurora, CO, USA). Dialysis bags with 500,000, 300,000, and 2000 Da retention capacities were purchased from MYM Biological Technology Company Limited (Chicago, IL, USA). Different Mw dextran standards (0.466 × 10^4^, 1.26 × 10^4^, 6.33 × 10^4^, 12.6 × 10^4^, 30.2 × 10^4^, and 55.6 × 10^4^ Da) were supplied by Chengdu Push Bio-technology Co., Ltd. (Chengdu, China). Monosaccharide standards (d-glucose anhydrous, d-fucose, d-galactose, d-xylose, d-arabinose, l-rhamnose, and d-mannose), erythritol, and glycerol were provided by Shanghai Ronghe Chemical Co., Ltd. (Shanghai, China). The protein concentration assay kit (Item No. P0012) was supplied by Beyotime Biotechnology Co., Ltd. (Shanghai, China). Pyridine, acetic anhydride, glacial acetic acid, HCl, NaOH, NaCl, 95% ethyl alcohol, NaNO_3_, and other reagents (AR-grade) were acquired from Sinopharm Chemical Reagent Co., Ltd. (Shanghai, China). Dulbecco’s modified eagle medium (DMEM) cell culture medium and phosphate buffered saline (PBS) buffer were provided by Wuhan Pricella Biotechnology Co., Ltd. (Wuhan, China). Lipopolysaccharide (LPS) was supplied by Sigma-Aldrich Co. (St. Louis, MO, USA). Fetal bovine serum (FBS) and penicillin-streptomycin were provided by GIBCO (Life Technology, New York, NY, USA). Dimethyl sulfoxide (DMSO) and methylthiazolyldiphenyl-tetrazolium bromide (MTT) were supplied by Beijing Solarbio Science & Technology Co., Ltd. (Beijing, China). The Ad12-SV40 2B transformed human bronchial epithelial cell line (BEAS-2B) was supplied by ATCC: The Global Bioresource Center (Manassas, VA, USA).

### 2.2. Extraction and Purification of ECP (Citri grandis fructus immaturus Polysaccharides)

Dried *Citri grandis fructus immaturus* was crushed and sieved. After degreasing with petroleum ether and anhydrous ethanol, crude powder was extracted via ultrasonic extraction in distilled water at 80 °C for 1 h, with a liquid-to-material ratio of 1:40 g/mL. Following filtration, the filtrate pressure was lowered and concentrated to 1/10, anhydrous ethanol was added to bring the ethanol concentration to 80%, and then after overnight ethanol precipitation at a low temperature, the filtrate residue was washed with anhydrous ethanol and ethyl acetate three times, re-solubilized in distilled water, and lyophilized to crude polysaccharide powder after removing insoluble matter. Finally, the Sevage approach was used to obtain the crude polysaccharides of *Citri grandis fructus immaturus* by removing proteins.

A crude polysaccharide solution of 30 mg/mL was formed by dissolving ECP in distilled water, and the solution was placed onto DEAE Sepharose fast flow anion exchange resin columns (3.5 cm × 60 cm) before being subjected to gradient elution using 0.1, 0.2, 0.3, and 0.4 mol/L NaCl solution at a flow rate of 2 mL/min. Using the phenol-sulfuric acid method, the concentration of polysaccharide in odd-numbered tubes was monitored, and the elution profile was plotted. Based on the elution profile, tubes 1–119, 120–179, 180–237, and 238–281 were combined. Next, the eluent was collected and concentrated and then dialyzed in a 2000 Da dialysis bag for 24 h to eliminate NaCl. After concentration and freeze-drying, four fractions (ECP1, ECP2, ECP3, and ECP4) of first-grade purified polysaccharides were obtained.

**Figure 1:**
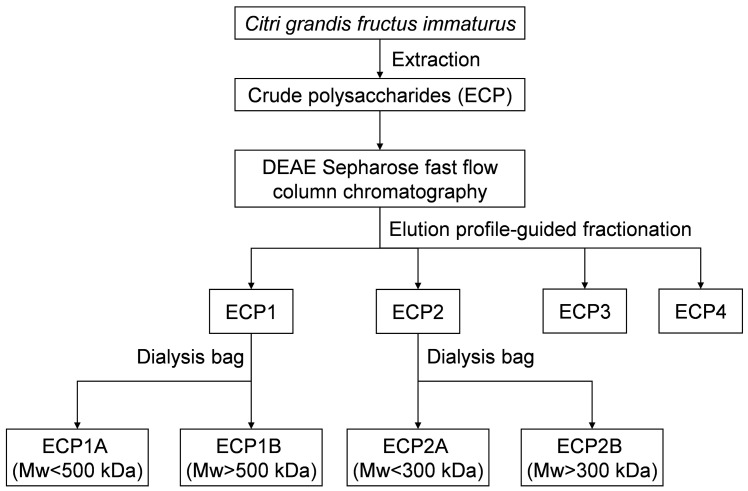
Extraction and purification procedure for *Citri grandis fructus immaturus* polysaccharides.

An appropriate amount of ECP1 was added to the purified water to form a 5 mg/mL polysaccharide solution, which was then dialyzed for 24 h in distilled water with the 500,000 Da dialysis bag. The process was repeated twice, and then the solution was combined and concentrated. ECP1A was the solution outside of the 500,000 Da dialysis bag, and ECP1B was the liquid within the bag. The operation of ECP1 was repeated, and ECP2 was purified with the 300,000 Da dialysis bag. ECP2A was the solution outside of the 300,000 Da dialysis bag, and ECP2B was the liquid inside the bag (Figure 1).

The protein contents of ECP1A, ECP1B, ECP2A, and ECP2B were assessed using a bicinchoninic acid (BCA) protein concentration assay kit. The meta-hydroxydiphenyl method [19] and phenol-sulfuric acid method [20] were used to quantify the glucuronide content and total sugar content of ECP1A, ECP1B, ECP2A, and ECP2B.

### 2.3. Characterization Analysis of Polysaccharides

#### 2.3.1. Triple Helix Structure Analysis

The approach used in this study is in line with You et al., with slight modifications [21]. A polysaccharide solution containing 5 mg/mL of ECP1A, ECP1B, ECP2A, and ECP2B was created by dissolving them in water. Then, 1 mL of 50 μg/mL Congo red solution was added, and 2 mol/mL NaOH solution was added progressively to raise the concentration of the solution to 0.18 mol/mL, 0.33 mol/mL, 0.46 mol/mL, 0.57 mol/mL, and 0.66 mol/mL. Following a 10-min room temperature equilibration period, β-glucan was used as the positive control, and pure water was the blank control. The samples in each tube were then scanned using UV spectroscopy in the 400–700 nm wavelength, and the maximum absorption wavelength (λmax) variations were recorded and compared.

#### 2.3.2. Monosaccharide Composition Analysis

With minor adjustments, the GC-MS analysis of the monosaccharide composition followed earlier results [22,23].

Four polysaccharide samples of *Citri grandis fructus immaturus* (5 mg) and monosaccharide standards were hydrolyzed with 2 mL of 4 mol/L trifluoroacetic acid (TFA) for 4 h at 110 °C. After the removal of TFA via concentration under reduced pressure, the hydrolysate was obtained by adding methanol and repeated dissolving and drying. After dissolving the hydrolysate with 2 mL of distilled water, sealing, and adding NaBH_4_, the reaction was shaken for 3 h at room temperature at 80 rpm. Glacial acetic acid was added, and the solution was shaken to remove excess NaBH_4_ and then evaporated with methanol several times under reduced pressure until all of the white powder was removed. The sample was acetylated by adding 3 mL acetic anhydride and 1 mL pyridine and sealed for 4 h at 100 °C. To obtain the acetylated product, the solution was cooled, concentrated, and dried multiple times using methanol under reduced pressure. After dissolving the acetylated product in 5 mL of chloroform, an equivalent amount of distilled water was added to extract it, the chloroform layer was recovered, and the process was repeated three times. Finally, anhydrous sodium sulfate was added to the chloroform layer, and it was shaken well and left overnight to remove residual water before being analyzed via GC-MS.

An Agilent 7890B GC-5977A MS gas chromatograph-mass spectrometer (Agilent, Santa Clara, CA, USA) equipped with a Thermo TG-35 ms quartz capillary column (30 m × 0.25 mm × 0.25 μm) was used for the GC-MS analysis. The temperature increase program was set as follows. The initial temperature was 95 °C, and the temperature was increased to 200 °C at a rate of 15 °C/min, 203 °C at 1.5 °C/min, 205 °C at 0.2 °C/min, 213 °C at 2 °C/min, 214 °C at 0.5 °C/min, and 234 °C at 15 °C/min. The carrier gas was helium, the injection port temperature was 270 °C without a shunt, the injection volume was 1 μL, and the flow rate was 1.0 mL/min. The bombardment source of mass spectrometry was EI; its scanning range was 35–450 amu, with an interface temperature of 250 °C, ion source temperature of 230 °C, and quadrupole temperature of 150 °C.

#### 2.3.3. Periodate Oxidation and Smith Degradation Analysis

Glycosidic linkage analysis was conducted using periodate oxidation-Smith degradation [24,25].

Exactly 15 mmol/L sodium periodate and sodium iodate solution were mixed according to a volume ratio (5:0, 4:1, 3:2, 2:3, 1:4, and 0:5), and 100 μL was diluted with water to 5 mL in a volumetric flask. The absorbance at 223 nm was measured with a UV spectrophotometer. Four polysaccharide samples (10 mg) were dissolved with 15 mmol/L sodium periodate and then diluted to 10 mL. The reaction was carried out at 4 °C and protected from light, and 20 μL of the solution was poured into a 5 mL volumetric flask at intervals of 2.5 h, 6.5 h, 18.5 h, and 24 h. Using distilled water as a blank control, the solution was diluted to the scale, and absorbance readings were measured at 223 nm until the absorbance variation ranged within ±0.02. Finally, periodate consumption was calculated. Formic acid production was calculated by adding 1% phenolphthalein test solution as an indicator to 3 mL of periodate-oxidized polysaccharide solution and titrating with 1 mmol NaOH solution.

To stop the periodate oxidation reaction, 1 mL of ethylene glycol was added to 5 mL of the residual reaction solution, and the mixture was allowed to sit for 2 h. After dialyzing the solution for 24 h in flowing water using a 2000 Da dialysis bag, it was dialyzed for another 24 h in distilled water. After concentrating the solution to 3 mL under decreased pressure and adding 70 mg of NaBH_4_, it was shaken for the entire night at 80 rpm. The solution was neutralized with 20% acetic acid, adjusting the pH down to 5–7. After 24 h of dialyzing with distilled water, the solution outside the dialysis bag was gathered and spun dry. After the solution in the dialysis bag was evaporated to dryness, complete acid hydrolysis and aldononitrile acetate derivatization were performed by adding 2 mol/L TFA solution. The Smith degradation products were analyzed via GC-MS using the following reference standards: glycerol, erythritol, l-rhamnose, d-arabinose, d-xylose, d-mannose, d-glucose, and d-galactose.

#### 2.3.4. Mw Determination

The dextran standards with Mw of 4, 16, 63, 120, 300, and 667 kDa were prepared as 8 mg/mL aqueous solutions and then injected into the high-performance gel permeation chromatography (HPGPC). The retention time of each sample was taken as the horizontal coordinate, and the log Mw was taken as the vertical coordinate. A polysaccharide solution containing 10 mg/mL was prepared by dissolving ECP1A, ECP1B, ECP2A, and ECP2B in distilled water and then analyzed via HPGPC. HPGPC analysis was conducted on a Shimadzu LC-20AT high-performance liquid chromatograph (HPLC) system (Shimadzu Corp., Kyoto, Japan) equipped with an Agilent PL aquagel-OH MIXED-H column (7.5 mm × 300 mm, 8 μm). The injection volume was 10 μL, and the mobile phase was 100% ultrapure water with a flow rate of 0.5 mL/min and a 15-min running time. The retention time was substituted into the standard curve to calculate the relative Mw of ECP1A, ECP1B, ECP2A, and ECP2B.

#### 2.3.5. FT-IR Spectra

Four *Citri grandis fructus immaturus* polysaccharide samples (5 mg) were fully dried and mixed with spectroscopic-grade KBr at 1:150 (*w*/*w*), ground, pressed into tablets, and then examined in the 400–4000 cm^−1^ wavelength region using an FT-IR spectrometer.

#### 2.3.6. Scanning Electron Microscopy (SEM)

A small amount of polysaccharide powder was completely dried and adhered to the sample stage, and after placing a conductive film of platinum in a vacuum spray plater, a LEO-1430-VP scanning electron microscope (Thermo Fischer, Waltham, MA, USA) operating at a high voltage of 20 KV was used to examine the polysaccharide’s exterior morphology.

### 2.4. Determination of Antioxidant Activity

#### 2.4.1. Assay of ABTS Radical Scavenging Activity

Four polysaccharides were tested for their capacity to scavenge ABTS radicals using established techniques with minor adjustments [26,27].

ECP1A and ECP2A were prepared as 2.00, 1.00, 0.40, 0.20, 0.04, and 0.02 mg/mL solutions of the samples to be tested. ECP1B and ECP2B were prepared as 5.00, 2.00, 1.00, 0.40, 0.20, and 0.10 mg/mL solutions of the samples to be tested. Different concentrations of the test solutions were added to 96-well plates (Table 1), and an enzyme-labeled instrument was used to detect the absorbance at 734 nm following uniform shaking after 6 min of reaction away from light. Distilled water was used as the negative control, and ascorbic acid was the positive control. Then, the scavenging effects of polysaccharides on ABTS free radicals were calculated.

#### 2.4.2. Impact of Polysaccharides on FRAP Oxidation Reactions

The effects of the four polysaccharides on FRAP oxidation reactions were implemented in accordance with a previous study with few modifications [28].

ECP1A, ECP1B, ECP2A, and ECP2B were prepared as 2.0, 1.0, 0.6, 0.2, 0.04, and 0.02 mg/mL of the test sample solution. Different concentrations of test solutions were added to the 96-well plate (Table 2), the absorbance values were measured at 593 nm using an enzyme-labeled instrument with ferrous sulfate as the positive control and distilled water as the negative control after shaking uniformly, and they were incubated for 10 min at a constant temperature of 37 °C away from light. The absorbance of the samples was substituted into the standard curve, and the FRAP values of the polysaccharides were calculated as Fe^2+^ antioxidant equivalents.

### 2.5. Measurement of Expectorant Activity

#### 2.5.1. Cell Viability Assay

BEAS-2B cells were cultured and proliferated with DMEM and passaged when they grew to 80–90%. BEAS-2B cells were cultivated overnight after being seeded at a density of 1 × 10^4^ cells/well in 96-well plates. After the cells had adhered to the wall in the monolayer, the supernatant was gently aspirated and discarded. After 24 h of stimulation with 100 μL of 2.0, 1.0, 0.5, 0.25, 0.125, and 0.0625 mg/mL polysaccharide sample solutions (ECP1A, ECP1B, ECP2A, and ECP2B), 10 μL of 5 mg/mL MTT solution was added. After 3 h of incubation, the supernatant was discarded, and 100 μL of DMSO was added. The solutions were shaken for 10 min, and the absorbance was measured at 490 nm on an enzyme-labeled instrument.

#### 2.5.2. Measurement of MUC5AC Mucin Content

BEAS-2B cells were seeded in 96-well plates at 1 × 10^4^ cells/ well and cultured for 24 h. The cells were randomly grouped; the blank control (normal cell culture), the model (treated with 5% cigarette smoke extract (CSE) combined with 200 ng/mL LPS in BEAS-2B cells for 24 h), and the drug groups (incubated for 24 h with the addition of low and high doses [0.125, 1.0 mg/mL] of ECP1A, ECP1B, ECP2A, and ECP2B, as well as 5% CSE and 200 ng/mL LPS, respectively). The low and high doses were determined based on cell viability assay and preliminary experiments. The MUC5AC mucin content in the supernatant of BEAS-2B cells was measured by ELISA.

### 2.6. Data Analysis

To determine the relationship between the structural characteristics of *Citri grandis fructus immaturus* polysaccharides and their antioxidant activity and expectorant activity, structural characteristics such as the Mw and monosaccharide composition were correlated with antioxidant activity and expectorant activity. One-way analysis of variance and correlation analysis were carried out using SPSS 27 software (IBM Company, Armonk, NY, USA) and plotted using GraphPad Prism 10 (GraphPad Software Inc., San Diego, CA, USA) and Origin 2021 software (OriginLab Corporation, Northampton, MA, USA).

## 3. Results

### 3.1. Extraction and Purification of Polysaccharides

**Figure 2:**
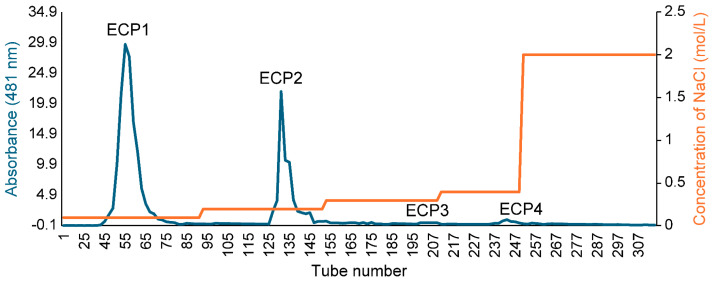
Elution profile of ECP via DEAE Sepharose.

The polysaccharide yield extracted from *Citri grandis fructus immaturus* was 15.6%. The ECP was purified with DEAE Sepharose fast flow exchange resin to obtain four primary purified polysaccharides (ECP1, ECP2, ECP3, and ECP4) (Figure 2). ECP1 and ECP2 showed higher transfer rates (24.55% and 33.72%, respectively). HPGPC revealed that ECP1 and ECP2 could be separated into two parts—from 500,000 and 300,000 Da—using dialysis bags with pure water, and four polysaccharides were obtained: ECP1A, ECP1B, ECP2A, and ECP2B.

### 3.2. Chemical Composition

The protein contents of ECP1A, ECP1B, and ECP2A were less than 5%, while the protein content of ECP2B was 15.23%, which may have been a proteoglycan (Table 3). The glucuronide contents of ECP1A, ECP1B, and ECP2A, which were neutral sugars, were 1.62%, 3.65%, and 2.43%, respectively, while the glucuronide content of ECP2B, which was an acidic polysaccharide, was 13.51%. ECP1A and ECP2A had low total sugar contents, whereas ECP1B and ECP2B had high total sugar contents of 73.11% and 28.47%, respectively.

### 3.3. Characterization of Polysaccharides

#### 3.3.1. Triple Helix Structure

Polysaccharide molecules with a single-stranded or multi-stranded helical space structure were able to form complexes with Congo red, and the λmax of the complexes was red shifted in comparison with that of Congo red. Also, under an alkaline environment, the complex structure was unwound, and a metastable region appeared, which manifested itself as a shift of the solution from red to purplish-red, along with a shift of the λmax in the long wave direction [29].

**Figure 3:**
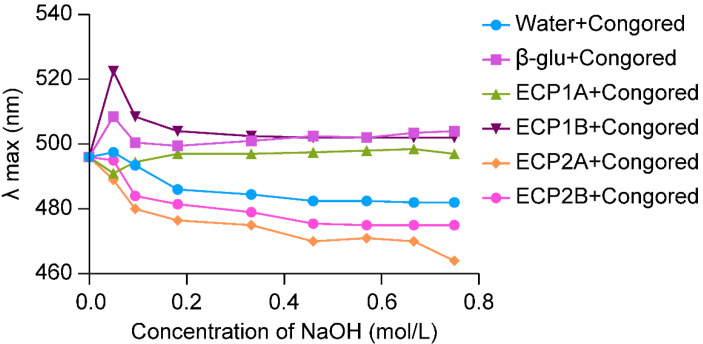
Triple helix structure analysis.

The λmax of the ECP1A-Congo red solution showed a significant red shift at low concentrations of alkali, and with an increase in the alkali concentration, the λmax increased briefly and then leveled off with a significant red shift (Figure 3). Based on previous studies [30,31], it can be confirmed that ECP1A is a single-stranded helix structure, which can be verified by more accurate experiments. The change in λmax of the ECP1B-Congo red solution was similar to that of the positive control group, both of which increased, decreased, and gradually leveled off, indicating that ECP1B’s spatial structure was similar to that of the positive control group, which might have been a three-stranded helical structure. The λmax of the ECP2A-Congo red solution and ECP2B-Congo red solution both increased weakly and decreased rapidly. This was insufficient to show that ECP2A and ECP2B had a triple-helical spatial structure because the maximum absorption wavelengths varied as the alkali concentration increased, and their change patterns were comparable to that of the blank control.

#### 3.3.2. Monosaccharide Composition

**Figure 4:**
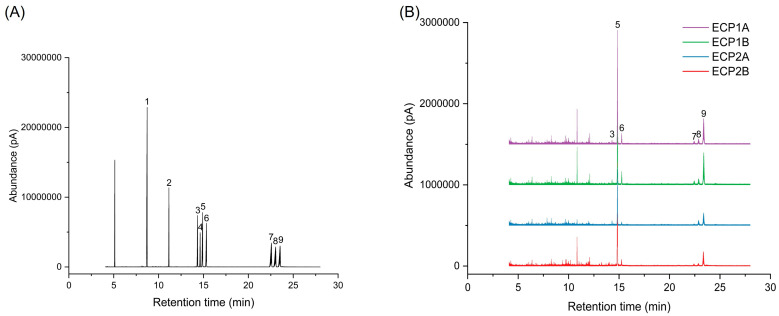
(**A**) Ion chromatogram of monosaccharide standard mixture and (**B**) ion chromatogram of four polysaccharides (1 = glycerol; 2 = erythritol; 3 = l-rhamnose; 4 = d-fucose; 5 = d-arabinose; 6 = d-xylose; 7 = d-mannose; 8 = d-glucose; 9 = d-galactose).

Figure 4 shows the gas phase-mass spectrometry chromatograms of the monosaccharide standard mixture and four polysaccharides after derivatization treatment. The concentration of standards is represented on the horizontal axis, with a peak area on the vertical axis to establish the monosaccharide standard curve. The chromatographic peaks of ECP1A, ECP1B, ECP2A, and ECP2B were substituted into the monosaccharide standard curve, and the results are shown in Table 4. It was shown that all four polysaccharides were composed of l-rhamnose, d-arabinose, d-xylose, d-mannose, d-glucose, and d-galactose. Among them, d-arabinose had the largest molar percentage (>50%), d-galactose had the second largest percentage, and d-mannose had the smallest molar percentage (<3%). ECP1A and ECP1B isolated from ECP1 had relatively similar monosaccharide composition structures, while the monosaccharide composition structures of ECP2A and ECP2B from ECP2 were significantly different, with a large difference in the overall composition, particularly for d-arabinose and d-glucose (Table 4).

#### 3.3.3. Periodate Oxidation and Smith Degradation

The absorbance values of ECP1A, ECP1B, ECP2A, and ECP2B reached stability after oxidation by periodate after 18.5 h, indicating complete reactions. According to the findings, 1 mol of hexose in ECP1A, ECP1B, ECP2A, and ECP2B consumed an average of 0.59 mol, 0.69 mol, 0.32 mol, and 0.64 mol of sodium periodate, respectively. The average amount of formic acid produced from 1 mol of hexose in ECP1B and ECP2B was 0.23 and 0.22 mol, respectively, indicating that ECP1B and ECP2B both contain a 1→6 glycosidic bond and a nonreducing terminal sugar group.

It has been reported that polysaccharides with 1→3, 1→4, and 1→2 or 1→6 glycosidic bonds generally produce glucose, erythritol, and glycerol, respectively, as the main products of Smith degradation reactions (Table 5) [32,33]. By comparing the GC chromatograms and experimental results, it was found that the products of polysaccharides after periodate oxidation and Smith degradation were divided into two major parts, and a large amount of propanetriol was detected in the inner portion of the dialysis bags of ECP1A and ECP1B, accounting for more than 70% of the contents. The amount of erythritol was smaller, and the proportion of the other monosaccharide compositions decreased substantially compared with those after periodate oxidation and Smith degradation, with a significant reduction in d-arabinose, indicating that d-arabinose in ECP1A and ECP1B may be 1→6 or 1→2 position-linked. The elevated percentage of glucose in the outer portion of the dialysis bag of ECP1A indicated a 1→6 glycosidic bond. The Smith degradation results were consistent with the results of the periodate oxidation reaction, indicating that the glycosyl groups in ECP1A and ECP1B were mostly linked by 1→2 or 1→6 glycosidic bonds, and a few were linked by 1→4 glycosidic bonds. Additionally, there were some glycosyl groups in ECP1A linked by 1→3 glycosidic bonds. ECP2A and ECP2B contained glycerol and erythritol, and a lower amount of erythritol was detected in ECP2A, while the compositional percentage of d-arabinose and d-galactose had minimal changes compared with those after periodate oxidation and Smith degradation, indicating that the d-arabinose and d-galactose in ECP2A and ECP2B may have been 1→3 linked. A large amount of glycerol was detected in the outer portion of the dialysis bag, indicating that the end of the main chain or the side chain of the *Citri grandis fructus immaturus* polysaccharides consisted of 1→2 or 1→6 linked sugar groups, and the outer portions of the dialysis bags of the ECP2A and ECP2B contained more glycerol and erythritol than those in the inner portions of the bags, indicating a higher branching degree compared with that of the ECP1A and ECP1B.

#### 3.3.4. Mw

**Figure 5:**
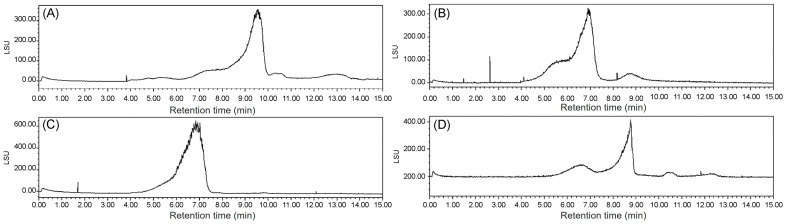
(**A**) Mw of ECP1A; (**B**) Mw of ECP1B; (**C**) Mw of ECP2A; and (**D**) Mw of ECP2B.

The Mw values of ECP1A, ECP1B, ECP2A, and ECP2B were determined via HPGPC with an evaporative light-scattering detector, where the retention time of each sample was substituted into the standard curve y = −1.0163 + 13.104, R^2^ = 0.9921, and the Mw values were calculated to be 340, 1048, 13, and 1217 kDa, respectively (Figure 5).

#### 3.3.5. FT-IR

**Figure 6:**
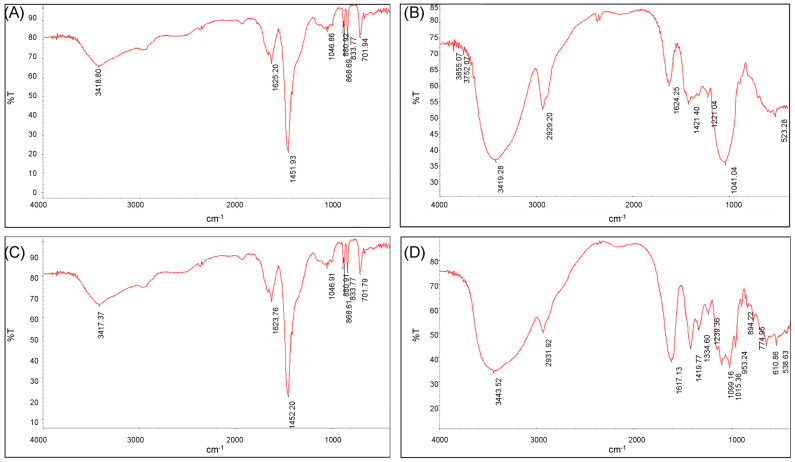
(**A**) FT-IR spectrum of ECP1A; (**B**) FT-IR spectrum of ECP1B; (**C**) FT-IR spectrum of ECP2A; and (**D**) FT-IR spectrum of ECP2B.

The scanned infrared spectra of ECP1A and ECP2A are shown in Figure 6A,C. The absorption peak at 2959 cm^−1^ was due to the C-H resonance of -CH_2_- on the sugar moiety [34,35], and the hydration resonance peaks on the sugar molecules caused the absorption peaks at 1625 cm^−1^ and 1623 cm^−1^ [36]. The C-O of the carboxyl group -COO on the sugar molecule caused the peaks at 1451 cm^−1^ and 1452 cm^−1^. The presence of a pyranose ring on ECP1A and ECP2A was demonstrated by a number of modest absorption peaks at 1000–1200 cm^−1^ [37,38], which are vibrational peaks caused by the C-O-C ether bond. The presence of a β-glycosidic bond was indicated by the absorption peak at 880 cm^−1^, while the presence of α-anomers was shown by the peak at 833 cm^−1^. The stacking ring stretching vibration of D-glucopyranose caused the absorption peak at 701 cm^−1^. There was no significant difference in the infrared spectral structures of the two polysaccharides.

The scanned infrared spectra of ECP1B and ECP2B were shown in Figure 6B,D. The O-H stretching vibration was responsible for the broad and large peaks at 3419 cm^−1^ and 3443 cm^−1^, while the C-H stretching vibration of -CH_2_- on the sugar moiety was responsible for the peaks at 2931 cm^−1^ and 2929 cm^−1^, and these two absorption peaks were the distinctive absorption peaks of the polysaccharide polymer compounds. The peaks at 1624 cm^−1^ and 1617 cm^−1^ were hydration resonance peaks on the sugar molecule; the C-O of carboxyl group -COO on the sugar molecule was the source of the peaks at 1421 cm^−1^ and 1419 cm^−1^; and the C-H angular vibration was responsible for the peaks at 1200–1400 cm^−1^. The strong and weak absorption peaks of ECP1B near 1040 cm^−1^ were bending vibrations of the C-O bond, indicating a furanose ring. The three characteristic peaks of ECP2B at 1000–1200 cm^−1^ indicated a pyranose ring, and the absorption peaks at 894 cm^−1^ and 774 cm^−1^ indicated a β-glycosidic bond.

#### 3.3.6. SEM

**Figure 7:**
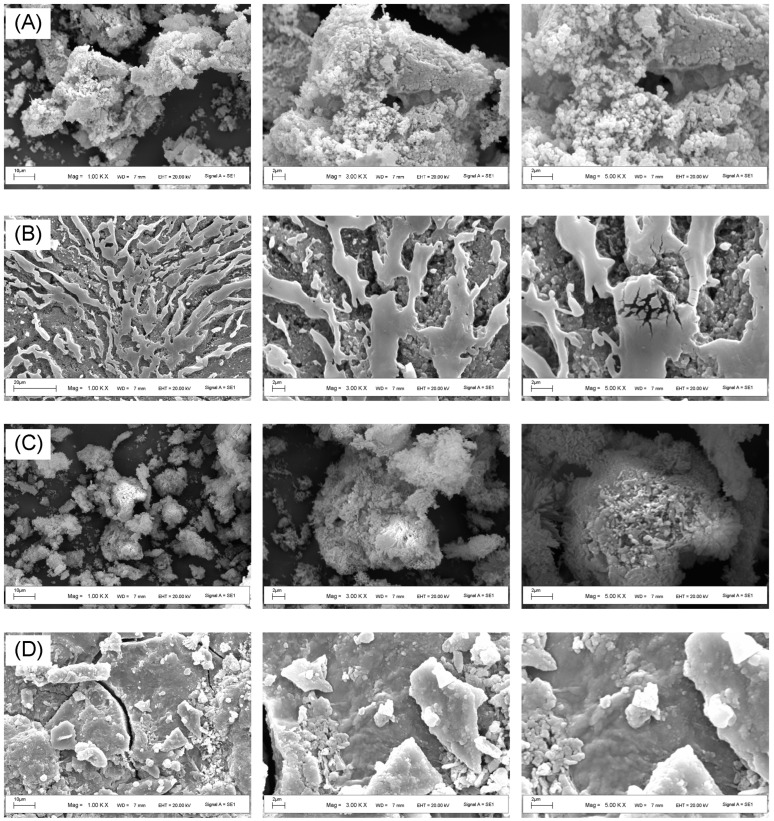
(**A**) SEM images of different parts of ECP1A; (**B**) ECP1B; (**C**) ECP2A; and (**D**) ECP2B.

ECP1A was a clustered polysaccharide with a spherical shape and a highly aggregated and loosely packed cotton-wool-like surface (Figure 7). ECP1B had a differentiated, flow-like multilayered structure with a concave-convex spherical bottom layer and a microfracture-type surface layer. ECP2A’s surface was rough and densely aggregated, with irregular mass and spherical aggregates visible at a high magnification. ECP2B had fine, linear veins and an irregular, slightly rough raised surface, with fine lines visible on the surface and irregular bulbous elevations elsewhere under a high magnification.

### 3.4. Antioxidant Activity

#### 3.4.1. ABTS Radical Scavenging Activity

**Figure 8:**
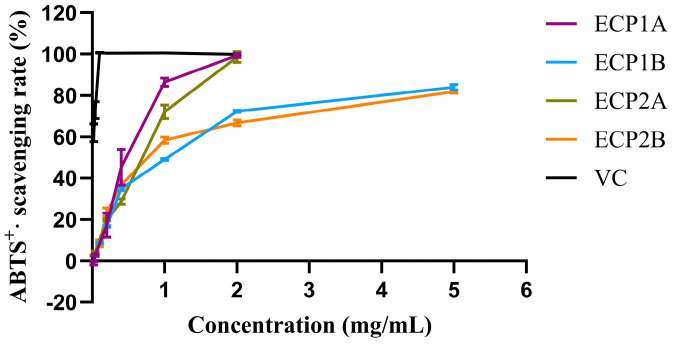
ABTS radical scavenging activity of polysaccharides (n = 3).

Among the four polysaccharides, ECP1A had the strongest antioxidant activity (IC_50_ = 0.4614 mg/mL), while ECP2A had the weakest ABTS radical scavenging activity (IC_50_ = 0.9448 mg/mL). The ABTS radical scavenging activities of the four polysaccharides increased gradually with increasing concentrations, with a consistent trend and slightly different growth rates (Figure 8). Overall, the scavenging activities of ECP1A and ECP2A were higher than those of ECP1B and ECP2B, indicating that the low-Mw polysaccharides had greater antioxidant activity than the high-Mw polysaccharides.

#### 3.4.2. Effect of Polysaccharides on FRAP Oxidation Reactions

**Figure 9:**
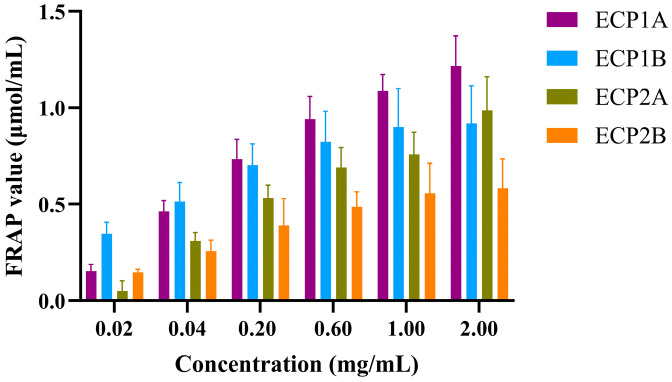
Effect of polysaccharides on FRAP oxidation reactions (n = 3).

The reducing activities of the four polysaccharides were dose-dependent, increasing with an increase in concentration (Figure 9). The rate of increase followed the order of ECP1A > ECP2A > ECP2B > ECP1B. At a concentration of 0.02 mg/mL, ECP1B had the strongest reducing activity, while ECP2A had the weakest activity. At concentrations > 0.2 mg/mL, ECP1A had the strongest activity, and ECP2B’s was the weakest.

### 3.5. Expectorant Activity

#### 3.5.1. Cell Viability

**Figure 10:**
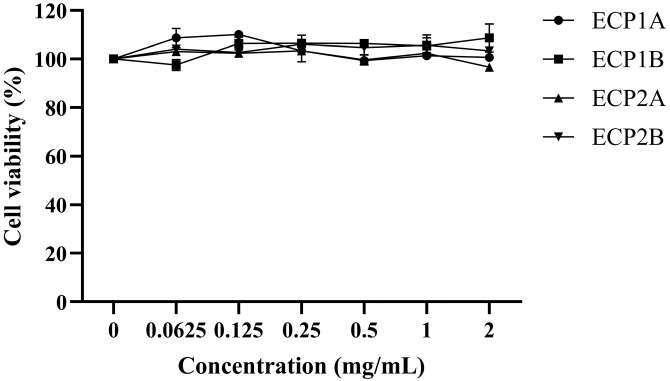
Effect of polysaccharides on the viability of BEAS-2B cells (n = 3).

The survival rate of the BEAS-2B cells treated with all four polysaccharides was approximately 100% in the concentration range of 0.0625–2.0 mg/mL (Figure 10). ECP1A, ECP1B, ECP2A, and ECP2B had no toxic effects on the BEAS-2B cells within the concentration range. Therefore, 0.125 and 1.0 mg/mL were chosen as the low and high doses in the subsequent experimental drug-dosing groups, respectively.

#### 3.5.2. MUC5AC Mucin Content

**Figure 11:**
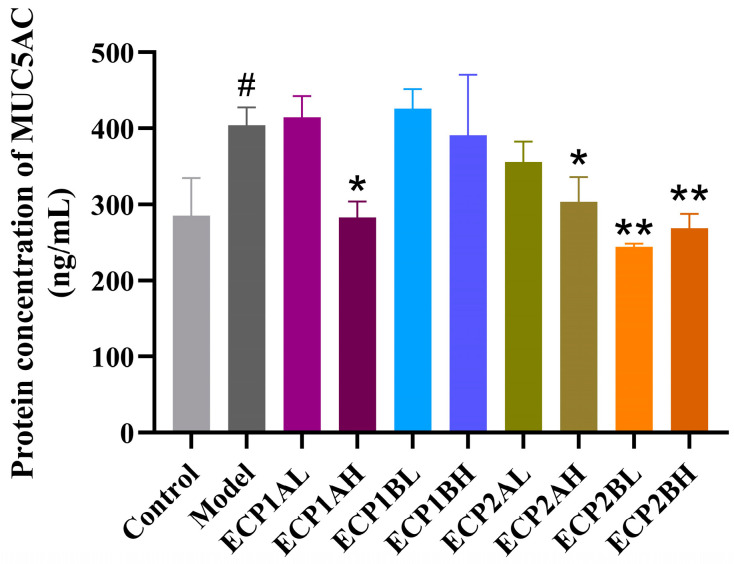
Effect of polysaccharides on MUC5AC mucin content expression level in BEAS-2B cells (n = 3). Control = blank group, Model = model group, L = low-dose group (0.125 mg/mL), H = high-dose group (1 mg/mL). ^#^ *p* < 0.05 compared with blank group. * *p* < 0.05 and ** *p* < 0.01 compared with model group.

ELISA was performed to determine the MUC5AC mucin content in the supernatant of BEAS-2B cells, and the results (Figure 11) showed that the MUC5AC mucin content was considerably higher in the model group than in the blank control group. In contrast to the model group, the MUC5AC mucin content expression levels in different drug-dosing groups decreased in a dose-dependent manner, indicating a better therapeutic effect from the high-dose group compared with the low-dose group. ECP2B had a relatively greater effect on MUC5AC mucin content expression in the BEAS-2B cells in the mucus hypersecretion model.

### 3.6. Correlation Analysis

**Figure 12:**
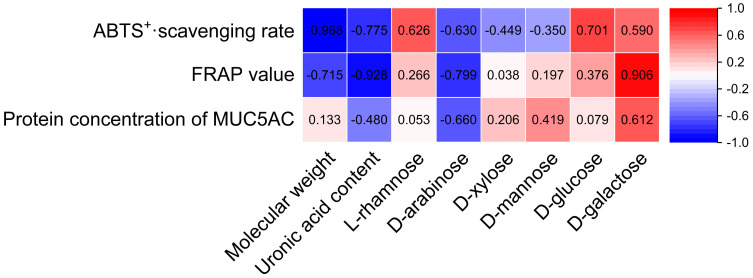
Heat map of the correlations between structural characteristics and activities of polysaccharides.

Structural characteristics such as the Mw, uronic acid contents, and monosaccharide compositions of *Citri grandis fructus immaturus* polysaccharides were correlated with their antioxidant and expectorant activities using SPSS 27 statistical software. As shown in Figure 12, the Mw, uronic acid content, d-arabinose, and d-galactose of *Citri grandis fructus immaturus* polysaccharides had strong correlations with its antioxidant and expectorant activities.

The Mw demonstrated a strong significant negative correlation with the ABTS^+^· scavenging rate (r = −0.968, *p* < 0.05) while showing a strong but non-significant negative trend with the FRAP value (r = −0.715, *p* > 0.05). Several other correlations showed notable trends without reaching statistical significance. The uronic acid content showed strong negative trends with the ABTS^+^· scavenging rate (r = −0.775) and FRAP value (r = −0.928), as well as a negative trend with the MUC5AC protein concentration, with a correlation coefficient of −0.480 (all *p* > 0.05). The d-arabinose content showed strong negative trends with the ABTS^+^· scavenging rate, FRAP value, and protein concentration of MUC5AC, with correlation coefficients of −0.630, −0.799, and −0.660 (*p* > 0.05 for all), respectively. The d-galactose content exhibited strong positive trends with the ABTS^+^· scavenging rate, FRAP value, and MUC5AC protein concentration, with correlation coefficients of 0.590, 0.906, and 0.612 (*p* > 0.05 for all), respectively.

## 4. Discussion

In this study, the four *Citri grandis fructus immaturus* polysaccharides were composed of six monosaccharides: l-rhamnose, d-arabinose, d-xylose, d-mannose, d-glucose, and d-galactose. In contrast, Cheng et al. identified *Citri Grandis Exocarpium* polysaccharide as a heteropolysaccharide consisting of d-xylose, d-glucose, d-galactose, l-arabinose, d-mannose, and an unknown monosaccharide, and infrared spectral analysis showed that it has α-glycosidic bond and glucopyranose ring [18], which is in line with the results of this study. The reason for the large difference in the composition and monosaccharide content may be due to the differences in the species, region, extraction method, and determination method of the raw materials used in the experiment.

The antioxidant and expectorant activities of the four *Citri grandis fructus immaturus* polysaccharides are closely related to the Mw, uronic acid content, and monosaccharide composition. In general, polysaccharides with extremely low Mw values are less likely to form active space structures, while polysaccharides with extremely high Mw values can hinder polysaccharides from exerting their biological activities in organisms, and polysaccharides with optimal Mw values will maximize their biological activity [39]. Que et al. revealed that among the four polysaccharide components of brown algal polysaccharides, the fraction with the lowest molecular weight exhibited the strongest antioxidant activity [40]. For the four *Citri grandis fructus immaturus* polysaccharides, the antioxidant activities of ECP1A and ECP2A, which had relatively low Mw values, were higher than those of the high-Mw polysaccharides. Pearson correlation analysis revealed a strong negative trend between the Mw values of *Citri grandis fructus immaturus* polysaccharides and their antioxidant activity. The uronic acid content order for the four polysaccharides was ECP2B > ECP1B > ECP2A > ECP1A, while the antioxidant activity order was ECP1A > ECP2A > ECP1B > ECP2B, which indicates that the antioxidant activity of *Citri grandis fructus immaturus* polysaccharides decreased with an increasing uronic acid content, which is consistent with AI’s findings [41]. The uronic acid contents of the *Citri grandis fructus immaturus* polysaccharides showed a strong negative correlation not only with its antioxidant activity but also with its expectorant activity. In a study by AI [41], it was reported that a higher arabinose content in tea polysaccharides reduced the antioxidant activity of tea polysaccharides. However, in this study, the ECP2B d-arabinose content was significantly higher than those of the other three polysaccharides, and its antioxidant activity was the poorest. Correlation analysis also confirmed that the d-arabinose contents of *Citri grandis fructus immaturus* polysaccharides showed strong negative trends with their antioxidant and expectorant activities.

Zhou et al. revealed that the preliminary antioxidative mechanism of crude sunflower disc polysaccharide involved the downregulation of Nrf2, HO-1, and NQO1 proteins in RAW264.7 cells [42]. ECP1A may similarly exert its antioxidant effects through the Nrf2/HO-1 pathways, although this hypothesis requires further experimental validation. Zhou [43] found that acidic polysaccharides from *Pyrus sinkiangensis* Yu. may exert anti-airway inflammation effects by interfering with the expression of NF-κB, p65, or MUC5AC, among others, in mouse lung tissue, thereby affecting the synthesis of key mRNAs of the NF-κB and MAPK signaling pathways as well as airway mucus secretion. In this study, ECP2B, an acidic polysaccharide from *Citri grandis fructus immaturus*, significantly inhibited MUC5AC mucin content expression in the mucus hypersecretion model of BEAS-2B cells induced by CSE combined with LPS. Huang et al. demonstrated that polysaccharides from alum-processed *Pinellia ternata* rhizomes significantly suppressed the expression of MUC5AC mRNA in the lung tissues of allergic asthma rats, consequently reducing mucus hypersecretion and phlegm accumulation, which accounts for their expectorant efficacy [44]. These findings suggest that the expectorant mechanism of ECP2B may involve the NF-κB/MAPK signaling pathways and regulation of mucin expression.

While this study provides valuable in vitro evidence for the antioxidant and expectorant activities of *Citri grandis fructus immaturus* polysaccharides, several limitations should be noted. The research relied on chemical-based assays and cellular models to evaluate the bioactivity of these polysaccharides. Furthermore, this study lacked in vivo validation using appropriate animal models. Most importantly, the mechanistic basis of the observed antioxidant and expectorant effects remains unclear, limiting insights into the structure–activity relationships of *Citri grandis fructus immaturus* polysaccharides. These limitations highlight the need for future comprehensive studies integrating animal experiments with multi-omics technologies to fully elucidate their therapeutic potential and mechanisms of action.

## 5. Conclusions

The structures of the four polysaccharides—ECP1A, ECP1B, ECP2A, and ECP2B—from *Citri grandis fructus immaturus* were elucidated, as well as their in vitro antioxidant and expectorant activities. Structural analysis showed that all four polysaccharides were composed of six monosaccharides, including l-rhamnose, d-arabinose, d-xylose, d-mannose, d-glucose, and d-galactose. ECP1A might be a neutral polysaccharide containing an α/β-glucopyranose ring with a single-stranded helical conformation, while ECP2B appeared to be an acidic polysaccharide composed of a β-glucopyranose ring. Their molecular weights were determined to be 340 and 1217 kDa, respectively. In vitro experiments revealed that ECP1A possessed excellent antioxidant activity, and ECP2B and high concentrations (1 mg/mL) of ECP1A and ECP2A exhibited significant expectorant activity. Pearson correlation analysis revealed a strong negative trend between the Mw of *Citri grandis fructus immaturus* polysaccharides and their antioxidant activities. Several factors contributed to the variations in the antioxidant and expectorant activities of the four polysaccharides, which could serve as a crucial guide for their application in the food and pharmaceutical industries. Further studies should utilize in vivo experiments to elucidate the mechanism underlying these activities.

## Figures and Tables

**Table 1:** ABTS antioxidant sample preparation.

	Positive Control	Samples	Sample Blank Background	Working Liquid Background
ABTS working liquid (μL)	180	180	0	180
95% ethanol (μL)	0	0	180	20
Sample solutions (μL)	0	20	20	0
Positive solution (μL)	20	0	0	0

**Table 2:** FRAP antioxidant sample preparation.

	Positive Control	Samples	Sample Blank Background	Working Liquid Background
FRAP working liquid (μL)	180	180	0	180
Distilled water (μL)	0	0	180	20
Sample solutions (μL)	0	20	20	0
Positive solution (μL)	20	0	0	0

**Table 3:** Chemical compositions of four secondary purified polysaccharides (n = 2).

	ECP1A	ECP1B	ECP2A	ECP2B
Protein (%)	3.54 ± 0.15	3.14 ± 0.14	4.42 ± 0.24	15.23 ± 0.37
Uronic acid (%)	1.62 ± 0.00	3.65 ± 0.00	2.43 ± 0.00	13.51 ± 0.00
Total sugar (%)	5.85 ± 0.00	73.11 ± 0.01	3.33 ± 0.00	28.47 ± 0.00

**Table 4:** Molar ratios of monosaccharide compositions of *Citri grandis fructus immaturus* polysaccharides (n = 2).

Monosaccharide	ECP1A	ECP1B	ECP2A	ECP2B
l-rhamnose	1.99 ± 0.01	2.01 ± 0.05	4.70 ± 0.26	1.54 ± 0.06
d-arabinose	52.38 ± 0.27	50.44 ± 1.62	50.20 ± 0.49	65.13 ± 1.34
d-xylose	6.99 ± 0.02	7.08 ± 0.21	3.77 ± 0.06	6.34 ± 0.32
d-mannose	2.64 ± 0.01	2.69 ± 0.11	2.31 ± 0.02	2.51 ± 0.01
d-glucose	5.15 ± 0.01	4.83 ± 0.21	9.66 ± 0.17	3.58 ± 0.03
d-galactose	31.15 ± 0.15	30.59 ± 1.16	28.42 ± 0.46	22.07 ± 0.12

**Table 5:** Molar ratio of periodate oxidation and Smith degradation products (n = 2).

	Outer Portions of Dialysis Bags				Inner Portions of Dialysis Bags			
	ECP1A	ECP1B	ECP2A	ECP2B	ECP1A	ECP1B	ECP2A	ECP2B
Glycerol	34.83 ± 1.09	64.28 ± 0.32	50.31 ± 0.23	41.94 ± 0.32	74.45 ± 0.20	76.33 ± 0.07	34.19 ± 0.22	23.22 ± 0.05
Erythritol	7.32 ± 2.12	10.31 ± 0.24	5.21 ± 0.64	3.06 ± 0.03	10.69 ± 0.06	3.94 ± 0.22	2.91 ± 0.03	16.26 ± 0.09
l-rhamnose	7.57 ± 0.22	2.44 ± 0.02	5.62 ± 0.07	2.31 ± 0.03	1.93 ± 0.01	1.48 ± 0.02	3.21 ± 0.13	5.08 ± 0.03
d-arabinose	3.48 ± 0.02	7.64 ± 0.01	7.71 ± 0.16	16.28 ± 0.07	1.85 ± 0.00	8.97 ± 0.01	21.40 ± 0.30	23.96 ± 0.07
d-xylose	4.93 ± 0.07	2.57 ± 0.02	4.18 ± 0.01	2.61 ± 0.03	1.55 ± 0.02	1.74 ± 0.01	4.78 ± 0.02	2.05 ± 0.01
d-mannose	12.10 ± 0.12	3.68 ± 0.02	8.52 ± 0.03	3.88 ± 0.09	2.67 ± 0.03	2.06 ± 0.02	6.65 ± 0.05	2.24 ± 0.01
d-glucose	11.30 ± 0.29	4.00 ± 0.00	8.09 ± 0.04	5.36 ± 0.11	2.68 ± 0.05	2.29 ± 0.04	8.17 ± 0.03	2.81 ± 0.00
d-galactose	18.48 ± 0.45	5.07 ± 0.02	10.37 ± 0.3	24.56 ± 0.11	4.18 ± 0.05	3.19 ± 0.08	18.68 ± 0.25	24.39 ± 0.07

## Data Availability

The original contributions presented in this study are included in the article.

## Abbreviations

The following abbreviations are used in this manuscript:

BCA	bicinchoninic acid
CSE	cigarette smoke extract
DEAE	diethylaminoethyl
DMEM	Dulbecco’s modified eagle medium
DMSO	dimethyl sulfoxide
ECP	*Citri grandis fructus immaturus* polysaccharides
FRAP	ferric ion-reducing antioxidant power
FT-IR	Fourier transform infrared
GC-MS	gas chromatography-mass spectrometry
HPGPC	high-performance gel permeation chromatography
HPLC	high-performance liquid chromatograph
LPS	lipopolysaccharide
MTT	methylthiazolyldiphenyl-tetrazolium bromide
Mw	molecular weight
PBS	Phosphate-buffered saline
SEM	scanning electron microscopy
TFA	trifluoroacetic acid
